# Pathway of PPAR-gamma coactivators in thermogenesis: a pivotal traditional Chinese medicine-associated target for individualized treatment of rheumatoid arthritis

**DOI:** 10.18632/oncotarget.7419

**Published:** 2016-02-16

**Authors:** Yanqiong Zhang, Xia Mao, Qiuyan Guo, Ming Bai, Bo Zhang, Chunfang Liu, Yanqun Sun, Shao Li, Na Lin

**Affiliations:** ^1^ Institute of Chinese Materia Medica, China Academy of Chinese Medical Sciences, Beijing 100700, China; ^2^ MOE Key Laboratory of Bioinformatics and Bioinformatics Division, TNLIST, Department of Automation, Tsinghua University, Beijing 100084, China; ^3^ Tianjin International Joint Academy of Biotechnology & Medicine, Tianjin 300457, China

**Keywords:** rheumatoid arthritis, traditional Chinese medicine syndrome, network pharmacology, PPAR-gamma, individualized treatment

## Abstract

Traditional Chinese medicine (TCM) syndromes have been regarded as the crucial clinical manifestations for individualized diagnosis and treatment of complex diseases, including rheumatoid arthritis (RA) and cancer. Especially, RA patients are classified into cold and hot syndromes with different clinical manifestations, interventions and molecular mechanisms. Better effectiveness of a classic cold syndrome-specific herbal formula Wu-tou decoction (WTD) has been achieved. To explore molecular mechanisms of syndrome-specific formulae is of great clinical significance to improve the effectiveness and pertinence of treatment for the complex diseases with personalized conditions. However, the scientific basis of WTD treatment on RA with the cold syndrome remains unclear. Here, we predicted the putative targets for composite compounds contained in WTD using drugCIPHER-CS and constructed a WTD herbs-putative targets-RA related genes network. Next, a list of major WTD targets was identified based on their topological features, including the degree, node betweenness, closeness and k-coreness in the above pharmacological network. Importantly, pathway enrichment analysis revealed that these major WTD targets were significantly associated with the pathway of peroxisome proliferator-activated receptor (PPAR)-gamma (PPAR-γ) coactivators in thermogenesis. These computational findings were subsequently verified by experiments on a rat model of collagen-induced arthritis (CIA) with cold or hot syndromes, and on human fibroblast-like synoviocytes-rheumatoid arthritis (HFLS-RA) cell line. In conclusion, the pathway of PPAR-γ coactivators in thermogenesis might be one of the potential pharmacological targets of WTD to alleviate RA with the TCM cold syndrome. These findings may open new avenues for designing individualized treatment regimens for RA patients.

## INTRODUCTION

Traditional Chinese Medicine (TCM), which is based on empirical applications and experience distilled over thousands of years, has become an essential component of the current medical system. TCM is distinguished by unique diagnostic and therapeutic theory, which places an emphasis on the regulation of the integrity of the human body and the interactions between human individuals and the environment [1]. Syndromes (also known as Zheng in Chinese) have been regarded as the crucial clinical manifestations for individualized diagnosis and treatment of complex diseases, including rheumatoid arthritis (RA) and cancer [2]. Developing individualized treatment for such complex diseases based on syndrome type would benefit the patients with personalized conditions. In this context, many Chinese herbal formulae are divided into two types: hot-cooling formulae used for the treatment of hot syndrome, and cold-warming formulae used for the treatment of cold syndrome. Marked efficacies have been achieved when the syndrome-specific formulae are used for patients’ management.

**Figure 1:**
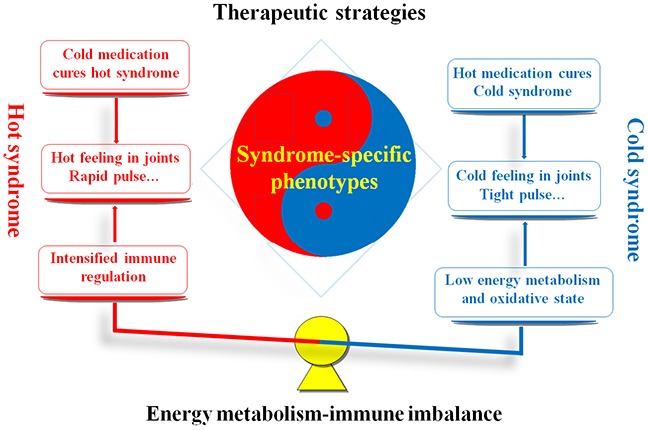
Similarities and differences between RA patients with hot and cold syndromes with regard to the clinical manifestations, therapeutic strategies and molecular mechanisms Red marks denote the hot syndrome, and blue marks denote the cold syndrome.

RA is categorized as an impediment disease (“Bi” syndrome) in TCM theory, which is caused by the invasion of wind, dampness or heat pathogens into the human body [3]. In the TCM clinical practice, RA patients are classified into cold and hot syndromes with different clinical manifestations, interventions and molecular mechanisms. As shown in Figure 1, RA patients with the cold syndrome are characterized by severe arthralgia which can be relieved by warming but aggravated by cooling, loose stools, an absence of thirst, clear profuse urine and a thin white tongue coating combined with a tight pulse. In contrast, RA patients with the hot syndrome suffer from severe arthralgia with red swelling of the skin and high skin temperature which can be relieved by cooling but aggravated by warming, constipation, thirst,deep-colored urine, a red tongue with a yellow coating, and rapid pulse [4]. In accordance to cold/hot syndromes, various pertinent TCM formulae have been created and used in China for thousands of years. Among them, Wutou decoction (WTD), Guizhi Fuzi decoction, and Yiyiren decoction are frequently used for the treatment of RA patients with cold syndrome. Many long-term clinical trials have revealed that the treatments of these formulae can efficiently alleviate symptoms and slow the course of the disease for approximately 90% of RA patients with cold syndrome [5–12]. Notably, the combination of these formulae with conventional medications such as nonsteroidal anti-inflammatory drugs (NSAIDs) and disease-modifying antirheumatic drugs (DMARDs) may have more satisfactory curative effects and reduce some side effects [13–16]. Moreover, Danggui Niantong decoction, Juanbi decoction, Sanshui Baihu decoction and Baihu Guizhi decoction are the primary prescriptions for RA patients with hot syndrome and nearly 80% of patients may achieve clinical remission after the administration of these formulae [17–23]. These data suggest that syndrome specific treatment may provide an efficient approach for RA patients in clinical management. In addition to clinical manifestations and interventions, understanding the molecular mechanism of syndromes is essential for syndrome differentiation and personalized therapy. Our previous studies constructed the molecular networks specific to cold syndrome and hot syndrome, and discovered the low level of energy metabolism in cold syndrome, which might be caused by the inhibition of nutrition intake and the decrease in the oxidative state. We also found that the immune regulation might be intensified in hot syndrome. These findings imply the associations between the energy metabolism-immune imbalance and cold/hot syndrome [24–25]. Peroxisome proliferator-activated receptor (PPAR)-gamma (PPAR-γ) coactivators, a group of nuclear transcriptional coactivators, play important roles in several metabolic processes including mitochondrial biogenesis, thermogenesis, respiration, insulin secretion and gluconeogenesis, and in regulation of inflammation [26–27]. The PPAR-γ agonists have also been regarded as promising therapeutic strategies for RA patients [28–29]. In this context, PPAR-γ coactivators involved into the modulation of energy metabolism and thermogenesis might be candidates for the treatment of RA patients with cold syndrome.

WTD, consisting of *Radix Aconiti, Herba Ephedrae, Radix Astragali, Raidix Paeoniae Alba* and *Radix Glycytthizae*, has been demonstrated to exert outstanding anti-inflammatory and analgesia effects [30]. Our previous studies have demonstrated the regulatory effects of WTD in the imbalance of nervous, endocrine and immune systems during RA progression [31, 32]. WTD has been adopted as a pertinent formula with hot nature for the treatment of RA patients with cold syndrome in clinics [5, 6]. It is extremely necessary to uncover the essence of hot nature of WTD and to explore the mechanism of WTD treatment on RA patients with the cold syndrome, in order to provide novel and efficient candidates for RA individualized treatment.

**Figure 2:**
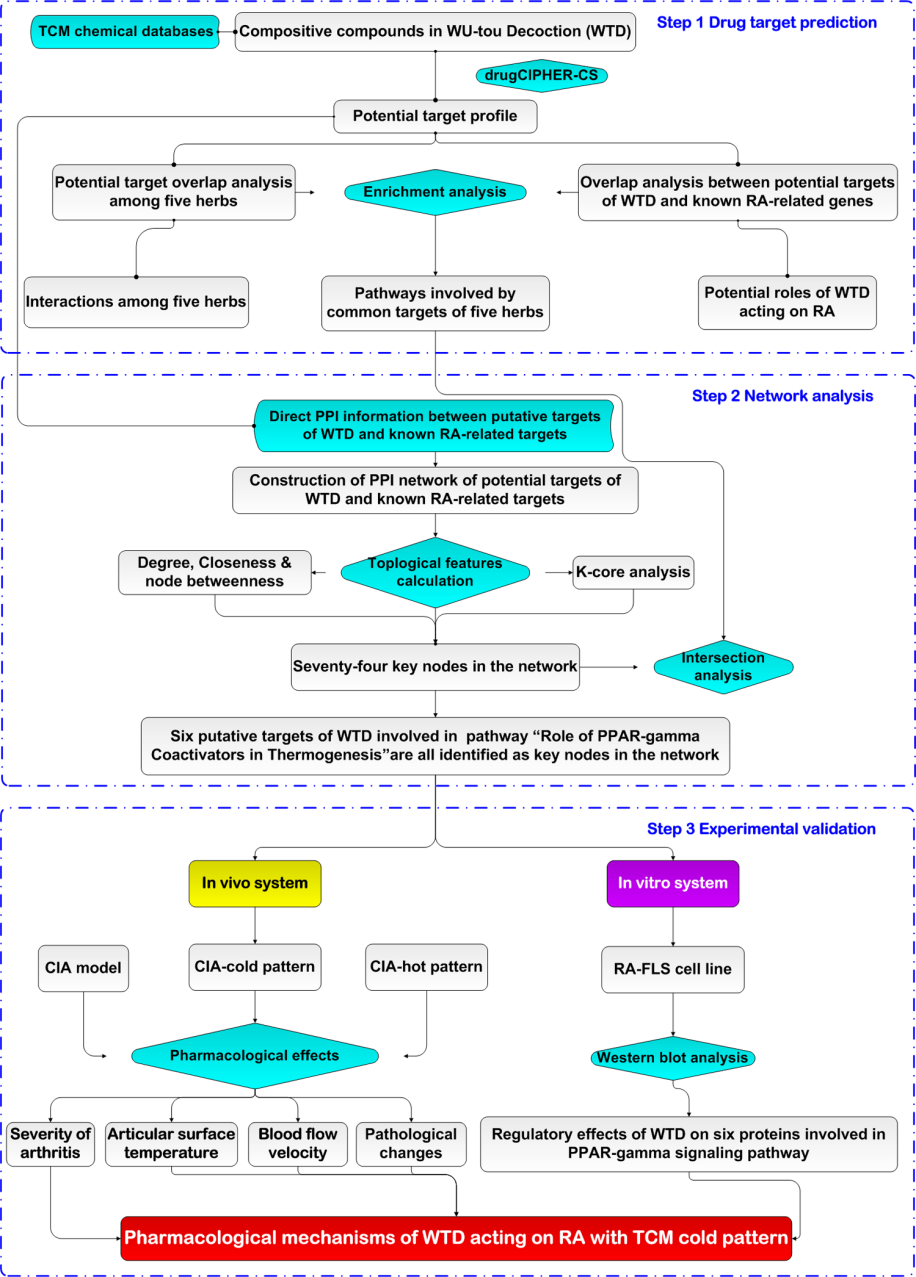
A schematic diagram of the systematic strategies for uncovering the pharmacological mechanisms of the herbal formula Wu-tou decoction acting on RA with the Traditional Chinese Medicine cold syndrome

Chinese herbal formulae with a large number of ingredients are too complex to be analyzed by traditional experimental methods based on the “one gene, one drug, one disease” paradigm. In recent years, the integration of TCM and network pharmacology, highlighting a “network target, multi-component therapeutics” paradigm, has provided an innovative perspective for the research of Chinese herbal formulae [33]. Therefore, the current study utilized a network pharmacology-based approach with the combination of computational analysis, animal and cell experiments to elucidate the underlying mechanisms of WTD acting on RA with cold syndrome. At first, we predicted the putative targets of WTD on the basis of the structures and functions of herbs contained in this formula; Then, the interaction network of WTD herbs-putative targets-RA-related genes was constructed. According to network analysis, topological comparison and literature mining, we found that the target genes related to the therapeutic effects of WTD on RA with cold syndrome might be mainly present in the pathway of PPAR-γ coactivators in thermogenesis. These computational findings were subsequently verified by experiments on a rat model of collagen-induced arthritis (CIA) with cold or hot syndromes, and on human fibroblast-like synoviocytes-rheumatoid arthritis (HFLS-RA) cell line. (Figure 2)

## RESULTS AND DISCUSSION

### Key putative targets of WTD acting on RA with the TCM cold syndrome

**Figure 3:**
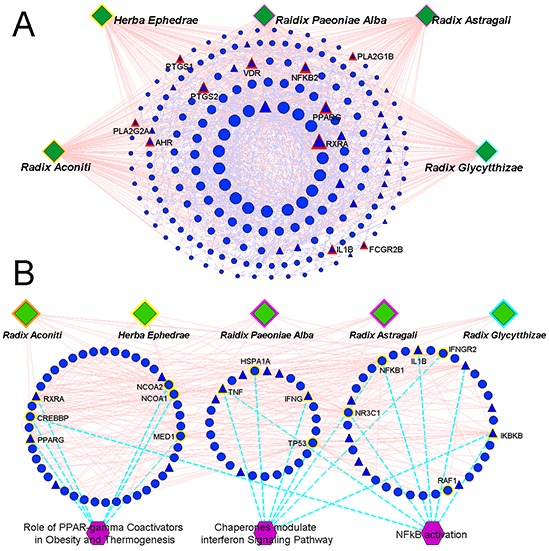
**A.** Network based on the direct interactions among five herbs (*Radix Aconiti, Herba Ephedrae, Radix Astragali, Radix Glycytthizae* and *Raidix Paeoniae Alba*), putative targets of WTD and known RA-related targets (herbs-putative targets-known RA targets network). Circular nodes refer to the putative targets of WTD; Triangle nodes refer to the known RA-related targets; Nodes with red highlights refer to the overlap between the putative targets of WTD and known RA-related targets; The size of the nodes were ordered according to their degree in the network. **B.** Network based on the direct interactions among the five herbs (*Radix Aconiti, Herba Ephedrae, Radix Astragali, Radix Glycytthizae* and *Raidix Paeoniae Alba*), major nodes of herbs-putative targets-known RA targets network and their associated pathways (herbs-major putative targets-pathway network). Nodes with yellow highlights refer to the major components involved into the corresponding pathways.

In our previous study [34], putative targets of WTD were predicted using the drugCIPHER-CS [35] (Supplementary Table S1). First, we constructed the network based on the direct interactions among herbs, putative targets of WTD and known RA-related targets (herbs-putative targets-known RA targets network). As shown in Figure 3A, this network consisted of 254 nodes: 5 herbs contained in WTD, 218 putative targets of this formula and 31 known RA-related targets. To screen the major nodes with topological importance, 4 topological features–“Degree,” “Node betweenness,” “Closeness” and “K value”–were calculated for each node in the network. The median values of “Degree,” “Node betweenness,” “Closeness” and “K value” were 5.00, 0.30, 8.13, and 4.00, respectively. Then, we determined that nodes with “Degree” >5.00, “Node betweenness” >0.30, “Closeness” >8.13, and “K value” >4.00 were major nodes. As a result, 74 major nodes–61 putative targets of WTD and 13 known RA-related targets–were identified (please see further detailed information on the topological features of the 74 major nodes in Supplementary Table S2).

According to the pathway enrichment analysis based on the Biocarta pathway database, the major nodes of the herbs-putative targets-known RA targets network were significantly associated with three pathways (Figure 3B): the “Role of PPAR-γ Coactivators in Obesity and Thermogenesis” (P_Bonferroni correction_=0.001), “Chaperones modulate interferon Signaling Pathway” (P_Bonferroni correction_=0.004) and “NF-κB activation” (P_Bonferroni correction_=0.036).

In TCM theory, WTD has hot herbal nature. Many long-term clinical trials have reported that this formula may exert better therapeutic effects on RA patients with the cold syndrome than those with the hot syndrome [5-6, 36-37]. Our research group previously performed a pharmacodynamics combined urinary metabolomics study using UPLC-Q-TOF-MS and found the decreased level of citric acid in the RA rat model, which could be reversed by the treatment of WTD [38]. Citric acid, produced in the tricarboxylic acid (TCA) cycle, has been indicated to be involved into energy metabolism. The serum level of citric acid is often reduced in bone diseases [39]. In this context, our previous data suggested that the energy metabolism might be broken down during RA progression, but could be restored by the administration of WTD. Additionally, our network-based investigations also discovered the low level of energy metabolism in cold syndrome [24–25]. In this study, our pathway enrichment analysis found that the major nodes of the herbs-putative targets-known RA targets network might be involved into the pathway of PPAR-γ coactivators in obesity and thermogenesis, which plays a crucial role in whole body metabolism by regulating adipocyte differentiation and energy storage [26–27]. Beyond this, PPAR-γ [target of Ibuprofen (DrugBank ID: DB01050, http://www.drugbank.ca/drugs/DB01050#bond-26699) and Indomethacin (DrugBank ID: DB00328, http://www.drugbank.ca/drugs/DB00328#bond-14736)] and RXRA [target of etodolac (DrugBank ID: DB00749, http://www.drugbank.ca/drugs/DB00749#bond-14722)] have been recognized as known therapeutic targets of RA. Growing evidence has also shown that PPAR-γ agonists may represent a new therapeutic approach for RA [40–42]. Thus, we hypothesized that the pathway of PPAR-γ coactivators in thermogenesis might be a potential target of WTD acting on RA with cold syndrome.

### Illustration of the pathway of PPAR-γ coactivators in obesity and thermogenesis

**Figure 4:**
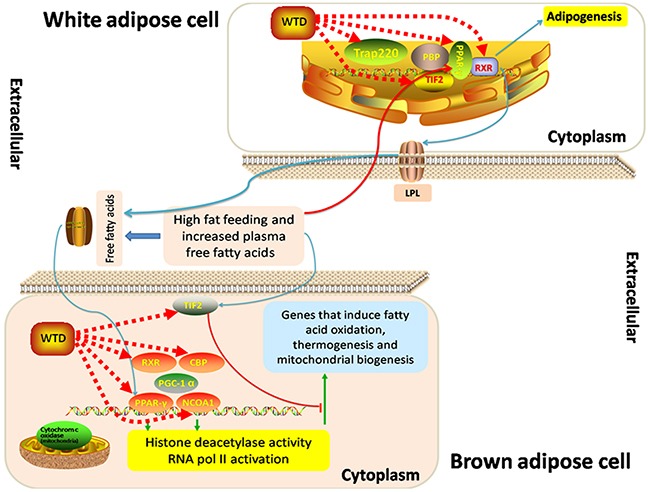
Schematic diagram of the “Role of PPAR-gamma Coactivators in Obesity and Thermogenesis.”

Six major nodes in the herbs-putative targets-known RA targets network (PPAR-γ, NCOA1, NCOA2, MED1, RXRA and CREBBP) are involved into the pathway of PPAR-γ coactivators in obesity and thermogenesis. They were all common putative targets for the five herbs contained in WTD with high reliability of drug target prediction according to the corresponding hit times and orders in putative target profiles (Table 1). As shown in Figure 4, PPAR-γ activation in brown adipose tissue depends mainly on Src-1 (NCOA1) as a coactivator, and Src-1 (NCOA1) is essential for PGC-1 alpha to act as a coactivator to stimulate thermogenesis. Regulation of energy use by PGC-1 in other tissues, such as muscle may contribute to weight loss associated with cancer and increased metabolism induced by exercise. In white adipose cells, Tif2 (NCOA2) predominates as the PPAR-γ coactivator, stimulating fat uptake and adipogenesis in conditions of high-fat feeding. Trap220 (MED1), a component of the TRAP coactivator complex, is also required for adipogenesis. Examining the metabolic consequences of coactivator deletion, Src-1 (NCOA1) and Tif2 (NCOA2) specifically alter PPAR-γ signaling. The change in the Tif2 (NCOA2) to Src-1 (NCOA1) ratio may also increase adipogenesis in white adipose, while blocking thermogenesis in brown adipose cells by opposing Src-1 (NCOA1)/PGC-1 alpha dependent signaling.

**Table 1:** Reliabilities of 6 putative WTD targets involved in the pathway of “Role of PPAR-γ Coactivators in Obesity and Thermogenesis” based on their hit times and orders in putative target profile

Five herbs (hit times/orders)					
	Radix Aconiti	Radix Astragali	Herba Ephedrae	Raidix Paeoniae Alba	Radix Glycytthizae
NCOA2	10(50)	32(5)	57(9)	48(7)	149(1)
NCOA1	18(2)	33(1)	63(2)	50(1)	148(3)
CREBBP	10(49)	30(9)	47(23)	44(12)	143(6)
RXRA	11(36)	22(14)	63(3)	49(4)	126(17)
MED1	18(1)	33(3)	62(4)	48(6)	148(4)
PPARG	4(196)	14(88)	48(20)	38(27)	115(40)

### Comparison of therapeutic effects of WTD on CIA rats with the cold syndrome and CIA rats with the hot syndrome

#### Severity of arthritis

**Figure 5:**
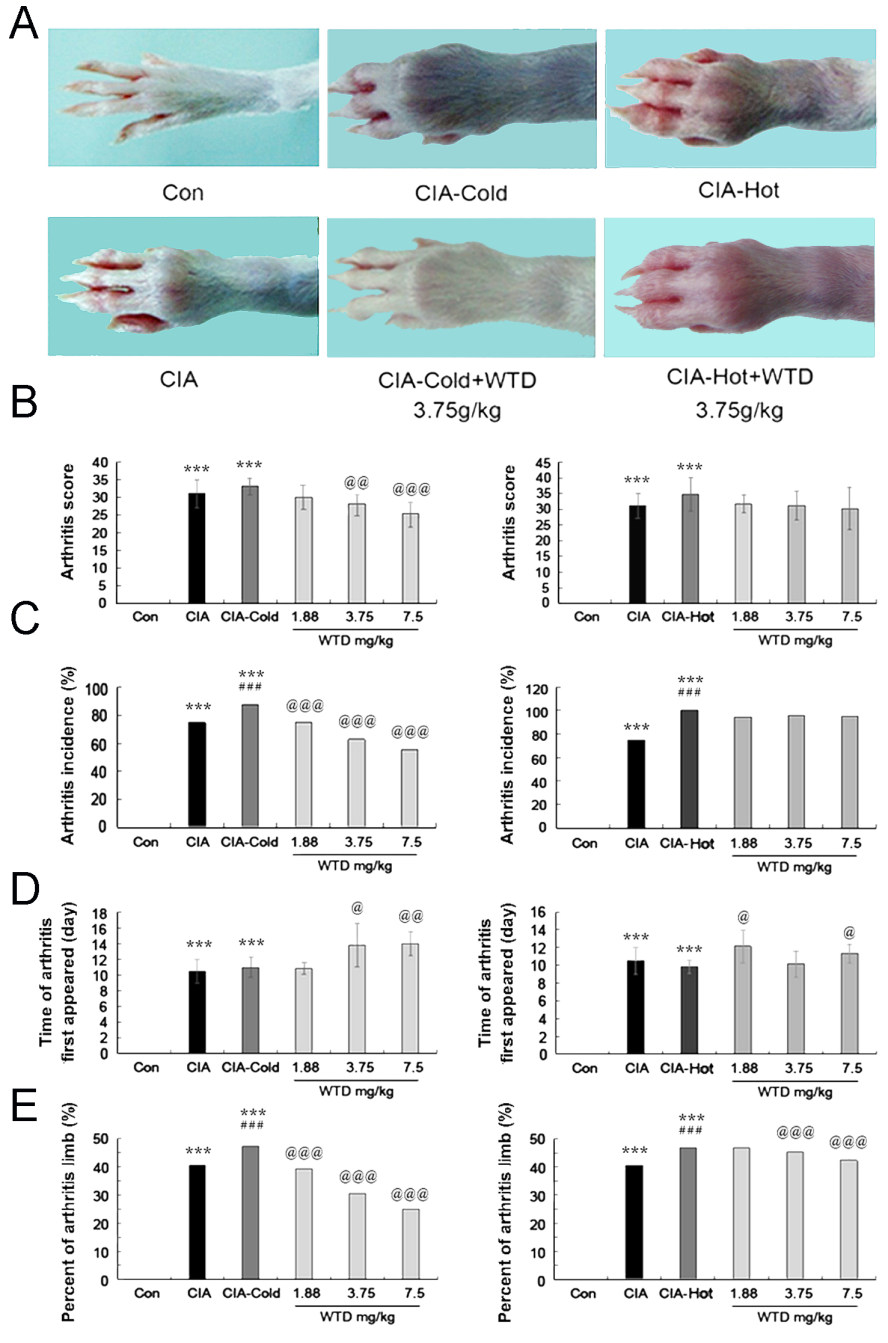
Effects of WTD on the severity of arthritis in CIA rats **A.** Macroscopic performance of arthritis, including erythema and swelling, was clearly observed in the CIA CIA-cold/hot model groups, whereas doses of 3.75 g/(kg•day) WTD significantly ameliorated the development and severity of arthritis in SD rats in the CIA-cold model groups; **B.** Doses of 3.75 and 7.5 g/(kg•day) WTD significantly decreased the mean arthritis score in the CIA-cold model group; **C.** Low-high doses of WTD significantly decreased the arthritis incidence in a dose-dependent manner in the CIA-cold model groups; **D.** Doses of 3.75 and 7.5 g/(kg•day) WTD significantly delayed the time when arthritis first appeared in the CIA-cold model groups; **E.** Low-high doses of WTD significantly decreased the percentage of arthritis limbs in the CIA-cold model groups; WTD could also decrease the severity of arthritis in CIA rats in the CIA-hot model group without statistical significance. Data are represented as the mean±S.D. (n=16). *, **, and ***, P<0.05, P<0.01, and P<0.001, comparison with the control group. ^#^, ^##^, ^###^, P<0.05, P<0.01, and P<0.001, comparison with the CIA model group. ^@^, ^@@^, ^@@@^, P<0.05, P<0.01, and P<0.001, comparison with the CIA-cold/hot model groups.

To investigate the therapeutic effects of WTD on arthritis, the CIA, CIA-cold and CIA-hot model in male SD rats were constructed. Although the manifestations of arthritis disease appeared at different times after immunization, we did not observe a relationship between the onset time of disease and clinical response. WTD was delivered by oral administration daily from the day when the SD rats were primary immunized and continued for a period of 21 days. As shown in Figure 5A, the macroscopic performance of arthritis, including erythema and swelling, was obviously observed in CIA, CIA-cold/-hot model groups, while doses of 3.75 g/(kg•day) WTD could significantly ameliorate the development and severity of arthritis in SD rats in CIA-cold groups. In addition, the mean arthritis score [for doses of 3.75 and 7.5 g/(kg•day), P<0.05, Figure 5B], arthritis incidence (all P<0.05, Figure 5C) and percentage of arthritic limbs (all P<0.05, Figure 5E) in WTD-treated rats, were significantly lower than those in the CIA-cold model group in a dose-dependent manner. Doses of 3.75 and 7.5 g/(kg•day) WTD also significantly delayed the time when arthritis first appeared in the CIA-cold rats (All P<0.05, Figure 5D). However, the therapeutic effects of WTD to the CIA-hot rats were not as prominent as those in WTD-treated CIA-cold groups. There were not any differences with statistical significance among the CIA-hot and CIA-hot-WTD treated groups (Figure 5A–5E).

In addition, we also detected the serum levels of the rheumatoid factors, IgG and IgM, in CIA rats, which are two isotypes of anti-cyclic citrullinated peptide antibody (anti-CCP) and are often used to pre-date the development of RA [43]. Compared to the control group, the serum levels of IgG and IgM were both increased in the CIA-cold/hot model groups (all P<0.05, Supplementary Figure S1A–S1B). Importantly, doses of 7.5 g/(kg•day) WTD significantly reduced the serum levels of IgG in both the CIA-cold/hot model groups (all P<0.05, Supplementary Figure S1A). Moreover, doses of 3.75 and 7.5 g/(kg•day) WTD (P<0.05, Supplementary Figure S1B) distinctly decreased the serum levels of IgM in the CIA-cold groups, while this decreasing tendency did not show a statistical significance in the CIA-hot groups.

#### Indicators of cold and hot syndromes

**Figure 6:**
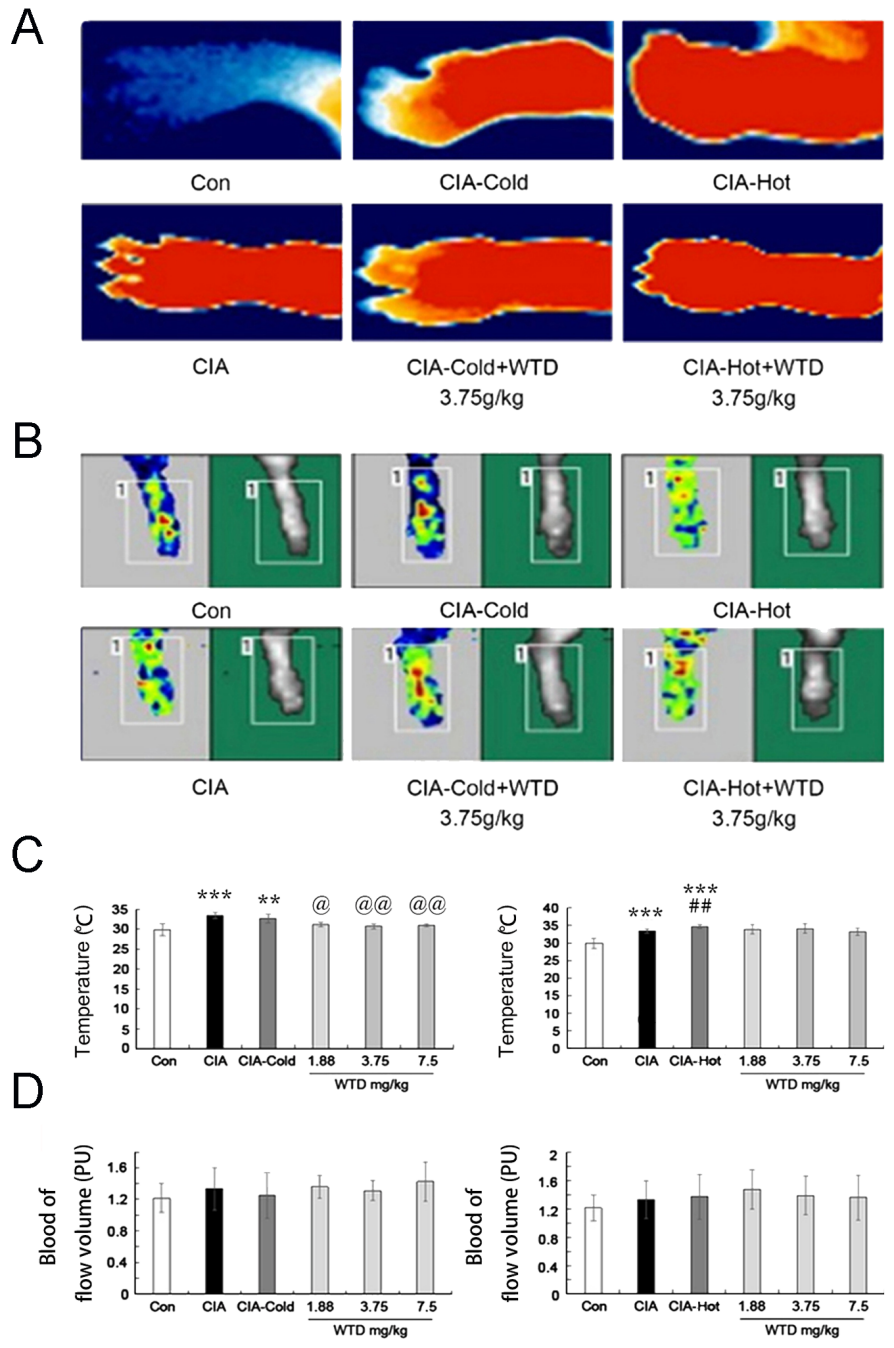
Effects of WTD on the temperature of the articular surface and the blood flow volume in CIA rats **A.** The temperature of the articular surface in CIA, CIA-cold/hot model groups were markedly increased compared with the control group, while doses of 3.75 g/(kg•day) WTD significantly decreased the temperature in both the CIA-cold /hot model groups; **B.** Blood flow volume in the joints tend to increase in the CIA, CIA-cold/hot model groups compared with the control groups, and doses of 3.75 g/(kg•day) WTD increased the blood flow volume in both the CIA-cold/hot model groups; **C.** The temperature of the articular surface were markedly up-regulated in the CIA, CIA-cold/hot model groups, which were markedly reversed by low-high doses of WTD in the CIA-cold groups. No statistical significance was observed in the CIA-hot model groups; **D.** WTD could increase the blood flow volume in the joints in both the CIA-cold/hot model groups without a significant difference. Data are represented as the mean±S.D (n=16). *, **, and ***, P<0.05, P<0.01, and P<0.001, comparison with the control group. ^#^, ^##^, ^###^, P<0.05, P<0.01, and P<0.001, comparison with the CIA model group. ^@^, ^@@^, ^@@@^, P<0.05, P<0.01, and P<0.001, comparison with the CIA-cold/hot model groups.

In this study, the temperature of the articular surface and blood flow volume in joints are regarded as two indicators to demonstrate the characteristics of RA cold and hot syndromes. Compared to the control groups, the temperature of the articular surface were markedly up-regulated in the CIA, CIA-cold/-hot model groups (all P<0.05, Figure 6A and 6C), which were markedly reversed by low-high doses of WTD in the CIA-cold groups (all P<0.05, Figure 6A and 6C). However, no differences with statistical significance were observed in the CIA-hot groups, although the same trend was observed in the CIA-cold groups (Figure 6A and 6C). In terms of blood flow volume in the joints, it has a tendency to increase in the CIA, CIA-cold/hot model groups compared with the control groups. Importantly, there were opposite tendencies between the CIA-cold (down) and CIA-hot (up) model groups against the CIA model group. WTD could increase the blood flow volume in joints in both CIA-cold/hot rats, although no significant difference was observed (Figure 6B and 6D).

#### Pathological changes

**Figure 7:**
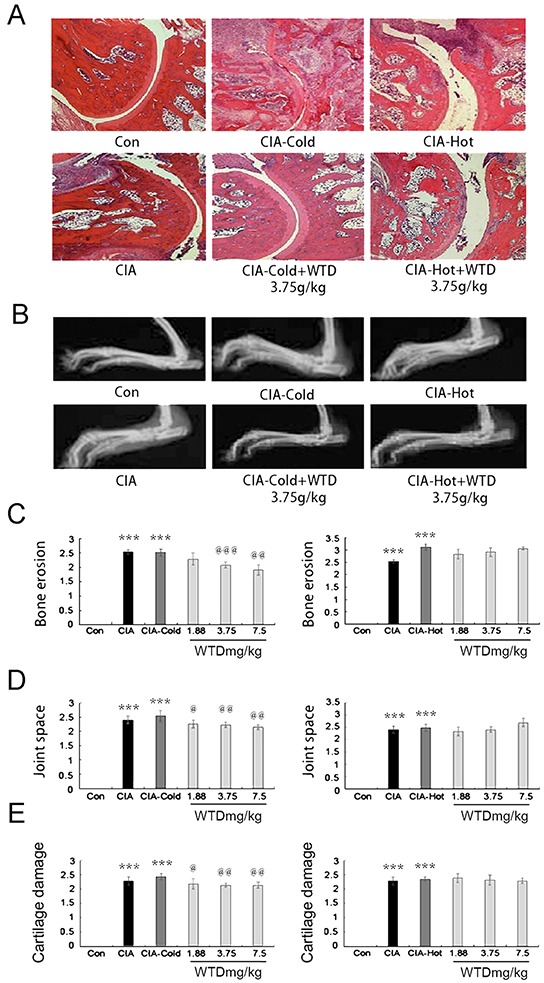
Effect of WTD on radiological changes and histologic lesions in CIA rats **A.** histologic observations of the joints in rats (HE staining); **B.** displays the clinical manifestation of CIA rats on day 21 after immunization, doses of 3.75 g/(kg•day) WTD improved paw swelling in the CIA-cold/hot model groups; **C. D.** and **E.** bone erosion, joint space and the degree of cartilage damage in joints, respectively, as described in the methods section. Data are represented as the mean±S.D (n=16). *, **, and ***, P<0.05, P<0.01, and P<0.001, comparison with the control group. ^#^, ^##^, ^###^, P<0.05, P<0.01, and P<0.001, comparison with the CIA model group. ^@^, ^@@^, ^@@@^, P<0.05, P<0.01, and P<0.001, comparison with the CIA-cold/hot model groups.

Radiological and histopathological evaluation of the ankle joints of SD rats in the CIA, CIA-cold/hot model groups revealed inflammatory cell infiltration, angiogenesis, synovial hyperplasia, synovial edema and joint damage. Oral administration of WTD could apparently attenuate the severity of the infiltration of the inflammatory cell, synovitis and bone damage, as well as narrow the joint space in the CIA-cold groups, while the therapeutic outcomes were not as evident as the outcomes observed in the CIA-hot groups. Normal synovial papillary structures were observed in the ankle section of SD rats in the control group without any inflammatory cells (Figure 7A and 7B). Statistically, we evaluated the anti-arthritis and joint protective effects of WTD on different groups of SD rats at the radiological and histologic level using semi-quantitative grading scales (on a scale of 0-3). The severity of bone erosion [for doses of 3.75 and 7.5 g/(kg•day), P<0.05, Figure 7C], joint space (all P<0.05, Figure 7D) and the degree of cartilage damage (all P<0.05, Figure 7E) were markedly decreased in a dose-dependent manner in the WTD-treated CIA-cold groups. However, no statistical significance was observed in the CIA-hot groups (Figure 7C–7E).

### WTD increases the expression of major components in the pathway of PPAR-γ coactivators in thermogenesis

**Figure 8:**
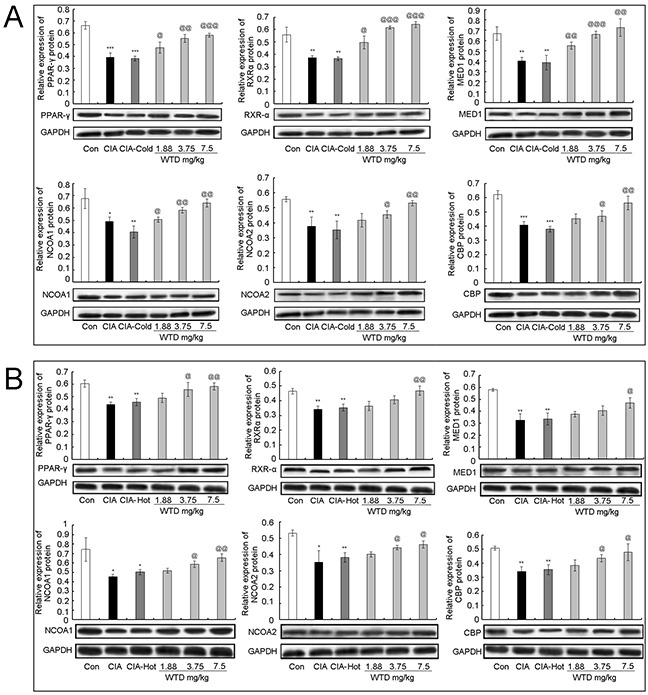
Effect of WTD on the expression of PPAR-γ **A.** RXR-α **B.** MED1 **C.** NCOA1 **D.** NCOA2 **E.** and CBP **F.** proteins in the joint part of CIA rats detected using western blotting analysis. Data are represented as the mean±S.D (n=16). *, **, and ***, P<0.05, P<0.01, and P<0.001, comparison with the control group. ^#^, ^##^, ^###^, P<0.05, P<0.01, and P<0.001, comparison with the CIA model group. ^@^, ^@@^, ^@@@^, P<0.05, P<0.01, and P<0.001, comparison with the CIA-cold/hot model groups.

**Figure 9:**
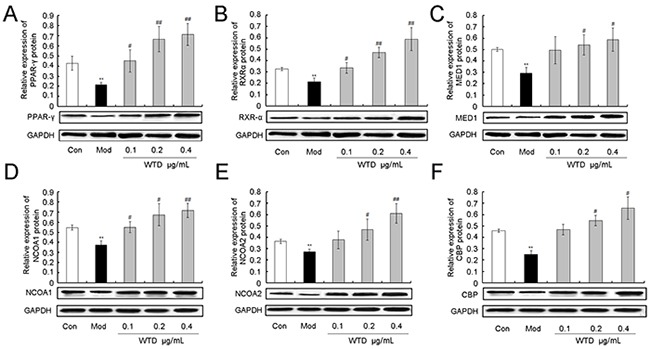
Effect of WTD on the expression of PPAR-γ **A.** RXR-α **B.** MED1 **C.** NCOA1 **D.** NCOA2 **E.** and CBP **F.** proteins in HFLS-RA. Data are represented as the mean±S.D. *, **, and ***, P<0.05, P<0.01, and P<0.001, comparison with the control group. ^#^, ^##^, ^###^, P<0.05, P<0.01, and P<0.001, comparison with the model group.

To investigate the pharmacological mechanisms of WTD on CIA with the cold syndrome, western blotting analysis was performed to detect the expression levels of NCOA1, NCOA2, RXR-α, CBP, PPAR-γ and MED1 proteins in the inflamed joints of CIA, CIA-cold/hot rats and HFLS-RA cells treated with various concentrations of WTD. Compared to the control groups, the expression levels of six proteins in the arthritis joints of CIA rats were dramatically decreased in the CIA, CIA-cold/hot model groups (Figure 8, all P<0.05), while the changes in the CIA-hot model group were less obvious than those in the CIA-cold model group. After the administration of WTD, the expression levels of PPAR-γ, RXR-α, MED1 and NCOA2 proteins were significantly increased in a dose-dependent manner in the CIA-cold groups (Figure 8A, all P<0.05). Doses of 3.75 and 7.5 g/(kg•day) WTD could enhance the expression of NCOA2 (both P<0.05) and CBP (both P<0.05) proteins in the CIA-cold groups. With regard to the CIA-hot groups, the upregulation effects of WTD on the six proteins were all weaker than those in the CIA-cold groups (Figure 8B). More importantly, these findings were all consistent with the data based on *in vitro* cultured HFLS-RA detected using western blotting analysis as shown in Figure 9.

## MATERIALS AND METHODS

### Drug target prediction for WTD

The putative targets of WTD's compositive compounds were predicted using drug CIPHER-CS as described in our previous study [34]. We provided this detailed information in Supplementary File S1-section 1.

### Network construction and analysis

We first constructed an interaction network for known RA-related targets (Supplementary File S1-section 2) and putative drug targets of WTD based on their direct interaction data obtained from eight existing PPI databases as described in Supplementary File S1-section 3. Next, we used Navigator software (Version 2.2.1) to visualize the interaction network.

Four measures–the degree, node betweeness, closeness and k-coreness–were calculated to assess the topological importance of the nodes in the network. The definitions of the four measures are provided in Supplementary File S1-section 4.

### Pathway enrichment analysis

We used the Database for Annotation, Visualization and Integrated Discovery [28] (DAVID, http://david.abcc.ncifcrf.gov/home.jsp, version 6.7) for pathway enrichment analysis based on pathway data obtained from Biocarta (http://www.biocarta.com/genes/index.asp). Only BioCarTa pathways with P-values <0.05 were included (both were corrected using the Bonferroni method).

### Experimental validation

The study was approved by the Research Ethics Committee of the Institute of Chinese Materia Medica, China Academy of Chinese Medical Sciences, Beijing, China. All animal studies were carried out in accordance with the guidelines and regulations for the care and use of laboratory animals of the Center for Laboratory Animal Care, China Academy of Chinese Medical Sciences.

### Preparation of WTD

The preparation of WTD was performed according to the original composition of this formula recorded in Chinese Pharmacopoeia 2010 edition [34]. Please see detailed information in Supplementary File S1-section 5.

### Animals

Male Sprague Dawley (SD) rats (n=100, 100 ± 5 g) were purchased from the Experimental Animal Center, Academy of Military Medical Sciences (production license no.: SCXK 2009-0017). All animals were maintained in a room with a constant temperature of 24 ± 1°C and with a 12-hour light/dark cycle, and allowed free access to food and water.

### Cell culture

In the current study, HFLS-RA (Cell Applications, San Diego, CA 92121, USA) were used for *in vitro* experimental validation. The cells were cultured in sterile synoviocyte growth medium (Cell Applications, San Diego, CA 92121, USA) containing 100 U/mL 1 penicillin, 80 U/mL 1 streptomycin, and 2 mM Gln-glutamine in a humidified atmosphere at 37°C in the presence of 5% CO_2_.

### Induction of CIA cold/hot model and treatment

For *in vivo* experimental validation, male SD rats were divided into 10 groups with 10 rats per group, which were separately categorized into the normal control group, the CIA model group, the CIA-hot model group, the CIA-hot+WTD-low/middle/high groups, the CIA-cold model group and the CIA-cold+ WTD-low/middle/high groups. Induction of the CIA model was performed as previously reported [44–46]. Briefly, male SD rats were injected intradermally at the base of the tail with 200 μg bovine type II collagen (Chondrex, Redmond, WA, USA) in 0.05 M acetic acid emulsified in incomplete Freund's adjuvant (IFA, Chondrex, Redmond, WA, USA). On day 7, rats were boosted intraperitoneally with 100 mg type II collagen in IFA. The first signs of inflammation were observed on day 11 after primary immunization. Induction of the CIA-cold/hot model was based on the sole induction of the CIA model. From the day of primary immunization, male SD rats were housed in the model box (production license no.: RXZ-380A) for two hours daily with specific wind velocity (6 m/s), temperature and humidity. Precisely, rats in the CIA-hot model group were retained in the box at 37°C and 95% humidity, while rats in the CIA-cold model group were housed in the box at 6°C and the 60% humidity. This process lasted for a period of 15 days. Treatment of WTD was performed for 21 days via oral administration from the first day, when the animals were primary immunized and the dosage selection for the low-, middle- and high-WTD were nearly equivalent to 0.5, 1 and 2 times of the daily dosage of RA patients, which were 1.88 g/kg, 3.75 g/kg and 7.5 g/kg, respectively.

For *in vitro* experimental validation, HFLS-RA cells were used at passage numbers between 4 to 8 and were incubated with 10 ng/mL of IL-1β and treated with various concentrations of WTD (0.1, 0.2 and 0.4 μg/mL) for 24 h.

### Severity assessment of arthritis

The severity of arthritis was assessed by arthritis score, percentage of arthritic limbs and the time of arthritis first appeared [34]. Please see detailed information in Supplementary File S1-section 6.

### Temperature of the articular surface

The temperature of the articular surface, awarded to the left hind paw of male SD rats, was measured using an Infrared thermal imager (TESTO-875, Testo AG, Schwarzwald, Germany) once a day from the day when the first signs of inflammatory were observed.

### Blood flow volume

The blood flow volume, awarded to the left hind paw of male SD rats, was measured using laser Doppler flowmetry (Moor VMS-LDF, Moor Instruments Ltd, Devon, UK) on day 22 after the first immunization.

### Histologic scoring

Histological changes among different groups were evaluated by two trained observers who were blinded to the background of the experiments, and were scored on the basis of joint space narrowing and cartilage damage of the ankle [34]. Please see detailed information in Supplementary File S1-section 7.

### Radiological observation

Radiographs of ankle and tarsus joints of each rat were evaluated for bone erosion using a semi-quantitative score system by two trained observers who were blinded to the background of the experiments [34]. Please see further detailed information in Supplementary File S1-section 8.

### Enzyme-linked immunosorbant assay (ELISA)

Sera were obtained from male SD rats on day 22 after the first immunization and reserved at −80°C until further use. The amount of IgG and IgM was detected using an ELISA (R&D Systems, Minneapolis, MN, USA) according to the manufacturer's protocol, and the absorbance was measured at 450 nm. All experiments were performed in triplicate.

### Western blotting analysis

To investigate the effect of WTD on the expression levels of six proteins–NCOA1, NCOA2, RXR-α, PPAR-γ, CBP, and MED1–both in the inflamed joints of different CIA rats and HFLS-RA cells treated with various concentrations of WTD, western blotting analysis was performed as described previously [44–47]. Following antibodies were used: NCOA1 antibody (rabbit monoclonal antibody, dilution 1:250, Sigma Aldrich, Saint Louis, MO, USA), NCOA2 (rabbit monoclonal antibody, dilution 1:300, Sigma Aldrich, Saint Louis, MO, USA), RXR-α antibody (rabbit polyclonal antibody, dilution 1:2500, Santa Cruz Biotechnology, Inc., Oregon, USA), PPAR-γ antibody (mouse monoclonal antibody, dilution 1:50, Santa Cruz Biotechnology, Inc., Oregon, USA), CBP antibody (rabbit monoclonal antibody, dilution 1:500, Cell Signaling, Boston, MA, USA), MED1 antibody (goat polyclonal antibody, dilution 1:50, Santa Cruz Biotechnology, Inc., Oregon, USA). All experiments were performed in triplicate.

### Statistical analysis

Statistical analyses were performed using SPSS statistical software (Version 13.0). Data are represented as the mean±S.D. The percentage of arthritic limbs was analyzed using the chi-square test. The arthritis index and pathological score were analyzed using non-parametric statistics (Kruskal-Wallis test). Other data were analyzed using one-way ANOVA followed by LSD test. *P*-values less than 0.05 were considered significant.

## CONCLUSIONS

WTD has a prominent therapeutic effect on the treatment of RA, particularly for RA patients with the cold syndrome, which best highlights the principle of “hot medication cures the cold syndrome” in TCM theory. In the current study, we proposed an integrative analysis that combines drug target prediction and network analysis, investigated the associations among herb-putative drug targets and disease gene-putative drug targets, and elucidated the pharmacological mechanisms of WTD on RA patients with the cold syndrome. Further experimental validations *in vitro* and *in vivo* provided convincing evidence that the pathway of PPAR-γ coactivators in thermogenesis might be one of the potential pharmacological targets of WTD to alleviate RA with the TCM cold syndrome. These findings may open new avenues for designing individualized treatment regimens for RA patients.

There are several potential limitations in our study. First, the insufficient evidence of how the precise ingredients contained in WTD affect putative targets might result in bias and the indeterminateness of this approach. Second, this work could not discriminate whether the interaction between a compositive component contained in this formula and the corresponding target are direct or indirect. Thus, additional studies are required in future work.

## SUPPLEMENTARY INFORMATION FIGURE AND TABLES












