# Supplementary material for: Pathway of PPAR-gamma coactivators in thermogenesis: a pivotal traditional Chinese medicine-associated target for individualized treatment of rheumatoid arthritis
Source: Oncotarget. 2016 Feb 16;7(13):15885–900. doi: 10.18632/oncotarget.7419 (PMC4941284; doi:10.18632/oncotarget.7419)
Supplement: Supplementary file 1 [file oncotarget-07-15885-s001.pdf]

SUPPLEMENTARY INFORMATION

Section 1. Drug target prediction for WTD

Composite compounds of each herb in WTD were obtained from TCM Database@Taiwan [1] (http://tcm.cmu.edu.tw/, Updated in 2012-06-28), which is currently the largest non-commercial TCM database worldwide. TCM Database@Taiwan is based on information collected from Chinese medical texts and scientific publications, and contains more than 20,000 pure compounds isolated from 453 TCM herbs. In total, we collected the structural information of 22 compounds for *Radix Aconiti*, 122 compounds for *Herba Ephedrae*, 39 compounds for *Radix Astragali*, 65 compounds for *Raidix Paeoniae Alba* and 203 compounds for *Radix Glycytthizae*. The detailed information on these compositive compounds of each herb in WTD is described in Supplementary Table S3.

The putative targets of WTD's compositive compounds were predicted by drug-CIPHER-CS presented by Zhao and Li [2]. Based on two hypotheses: (i) drugs with similar chemical structure usually bind functionally related proteins and (ii) functional relationship between the proteins can be measured by their distance in the protein interaction network, drugCIPHER-CS achieves good prediction performance and can infer drug targets in the genome-wide scale. This method calculates the likelihood of the interactions of drug-target based on the correlation between the query drug's structure similarity vector with the drug space and the candidate gene's functional similarity vector with the target space. For a query compound, drug-CIPHER-CS prioritizes the proteins in the PPI network according to the order of the decreasing drug target interaction likelihood, and the candidate proteins with high likelihood will be hypothesized as the putative targets.

Section 2. Known RA-related targets

Known RA-related targets were obtained from four existing resources: (1) DrugBank database [3] (http://www.drugbank.ca/, version: 3.0). We only used those drug-target interactions whose drugs are FDA approved for the treatment of RA and whose targets are human genes/proteins. In total, we obtained 58 known RA-related targets. (2) The Online Mendelian Inheritance in Man (OMIM) database [4] (http://www.omim.org/, Last updated: October 31, 2013). We searched the OMIM database with a keyword "rheumatoid arthritis" and found 7 known RA-related targets: CD244, HLA-DR1B, MHC2TA, NFKBIL1, PAD, SLC22A4, and PTPN8. (3) Genetic Association Database (GAD) [5] (http://geneticassociationdb.nih.gov/, Last updated: August 18, 2013). We used a keyword "rheumatoid arthritis" to search the GAD database. In total, we obtained 82 known RA-related targets whose association with RA was shown "Y". (4) Kyoto Encyclopedia of Genes and Genomes (KEGG) Pathway Database [6] (http://www.genome.jp/kegg/, Last updated: October 16, 2012). In total, we obtained 92 known RA-related targets which appear on the RA pathway (KEGG ID: map05323) in the KEGG database. The detailed information on these known therapeutic targets is described in Supplementary Table S4. After deleting redundancy, there were 208 known RA-related targets collected in this study.

Section 3. Protein-protein interaction (PPI) data

PPI data were imported from eight existing PPI databases including Human Annotated and Predicted Protein Interaction Database (HAPPI) [7], Reactome [8], Online Predicted Human Interaction Database (OPHID) [9], InAct [10], Human Protein Reference Database (HPRD) [11], Molecular interaction Database (MINT) [12], Database of Interacting Proteins (DIP) [13], and PDZBase [14]. The detailed information on these PPI databases is described in Supplementary Table S5.

Section 4. Defining network topological feature set

For each node i in interaction network, we defined four measures for assessing its topological property: (1) 'Degree' is defined as the number of links to node i; (2) 'Node betweenness' is defined as the number of shortest paths between pairs of nodes that run through node i. (3) 'Closeness' is defined as the inverse of the farness which is the sum of node i distances to all other nodes. The Closeness centrality can be regarded as a measure of how long it will take to spread information from node i to all other nodes sequentially. Degree, node betweenness and closeness centralities can measure a node's topological importance in the network. The larger a node's degree/ node betweenness /closeness centrality is, the more important the node is in the interaction network [15]. (4) K-core analysis is an iterative process in which the nodes are removed from the networks in order of least-connected [16]. The core of maximum order is defined as the main core or the highest k-core of the network. A k-core sub-network of the original network can be generated by recursively deleting vertices from the network whose degree is less than k. This results in a series of sub-

networks that gradually reveal the globally central region of the original network. On this basis, 'K value' is used to measure the centrality of node i.

Section 5. Preparation of WTD

According to the original composition of WTD recorded in Chinese Pharmacopoeia 2010 edition, WTD was prepared using the following procedure. The crude drugs of *Radix Aconiti* 6g, *Herba Ephedrae* 9g, *Radix Astragali* 9g, *Raidix Paeoniae Alba* 9 g and *Radix Glycytthizae* 9 g were immersed in 2 litres of water for 2 h and then decocted to boil for 1 h. The decoction was filtered through four layers of gauze. Next, the drugs were boiled once again for 0.5 h with 2litres of water and the decoction was filtrated out with the above method. Finally, the extraction solution was made to a concentration of 1 g crude drug/mL. To clarify the chemical composition of WTD, UPLC–Q-TOF-MS analysis was conducted to identify its major compounds. The detailed strategy and results of the identification were provided in our previously published paper [17].

Section 6. Severity assessment of arthritis

Rats were observed once every day after primary immunization. Arthritis severity was evaluated by arthritis score, percentage of arthritic limbs and the time of arthritis first appeared which were performed by two independent, blinded observers. The arthritis score was the total of the scores for all 4 limbs (maximum possible arthritis score 80). Arthritis incidence values are the number positive/total number in group. In addition, the number of arthritic limbs of individual rats were counted and added to represent the number of arthritic limbs in a group. The percentage of arthritic limbs in a group was calculated as following formula:

$$\text{Percentage of arthritic limbs} = \frac{\text{Number of arthritic limbs in a group}}{\text{Number of all limbs in a group}} \times 100\%$$

Moreover, the time of arthritis first appeared referred to the first day of the onset of the clinical symptoms of arthritis observed.

Section 7. Histology and histologic scoring

Rats were sacrificed by cervical dislocation on day 21 after first immunization. Both hind limbs including the paws, ankles, and knees, were dissected, fixed immediately for 24 h in 4% paraformaldehyde, decalcified in 10% EDTA for up to 2 month at 4°C, and embedded in paraffin. Tissue sections (4mm) were mounted on common slides for staining with hematoxylin and eosin (H&E). Histological observations were graded on the basis of joint space narrowing and cartilage damage of the ankle on a scale of 0 (normal), 1 (mild changes), 2 (moderate changes), and 3 (severe changes) by two trained observers who were blinded to the treatment groups. Histopathologic scores were expressed as the summation of the scores awarded to the left hind paw by both observers, with a maximum score of 6 per rat for each histologic parameter. Minor differences between observers were resolved by mutual agreement [18, 19].

Section 8. Radiological observation

At the end of the experiment, rats were sacrificed and the left hind paws were radiographed with a digital mammography system (Planmed, Finland). Radiographs of ankle and tarsus joints of each rat were evaluated for bone erosion using a semiquantitative scale of 0=normal, 1=mild changes, 2=moderate changes, and 3=severe changes, respectively [20]. Two observers blind to treatment assignment and with significant experience in reading and rating radiographs for patients with RA evaluated the radiographs. A total radiological score was obtained by summing the scores awarded to the left hind paw by both observers, giving a maximum score of 6 per rat for each radiological parameter.

REFERENCES

1. Chen CY. TCM Database@Taiwan: The World's Largest Traditional Chinese Medicine Database for Drug Screening In Silico. PLoS One. 2011; 6:e15939.
2. Zhao S, Li S. Network-based relating pharmacological and genomic spaces for drug target identification. PLoS One. 2010; 5:e11764.
3. Wishart DS, Knox C, Guo AC, Cheng D, Shrivastava S, Tzur D, Gautam B, Hassanali M. DrugBank: a knowledgebase for drugs, drug actions and drug targets. Nucleic Acids Res. 2008; 36:D901-6.
4. Hamosh A, Scott AF, Amberger JS, Bocchini CA, McKusick VA. Online Mendelian Inheritance in Man (OMIM), a knowledgebase of human genes and genetic disorders. Nucleic Acids Res. 2005; 33:D514-7.
5. Becker KG, Barnes KC, Bright TJ, Wang SA. The genetic association database. Nat Genet. 2004; 36:431-2.
6. Wixon J, Kell D. The Kyoto encyclopedia of genes and genomes–KEGG. Yeast. 2000; 17:48-55.

7. Chen JY, Mamidipalli S, Huan T.HAPPI: an online database of comprehensive human annotated and predicted protein interactions. BMC Genomics. 2009; 10: S16
8. Matthews L, Gopinath G, Gillespie M, Caudy M, Croft D, de Bono B, Garapati P, Hemish J, Hermjakob H, Jassal B, Kanapin A, Lewis S, Mahajan S, May B, Schmidt E, Vastrik I, Wu G, Birney E, Stein L, D'Eustachio P. Reactome knowledgebase of human biological pathways and processes. Nucleic Acids Res. 2009; 37:D619-22.
9. Brown KR, Jurisica I. Online predicted human interaction database. Bioinformatics. 2005; 21:2076-82.
10. Aranda B, Achuthan P, Alam-Faruque Y, Armean I, Bridge A, Derow C, Feuermann M, Ghanbarian AT, Kerrien S, Khadake J, Kerssemakers J, Leroy C, Menden M, Michaut M, Montecchi-Palazzi L, Neuhauser SN, Orchard S, Perreau V, Roechert B, van Eijk K, Hermjakob H. The IntAct molecular interaction database in 2010. Nucleic Acids Res. 2010; 38:D525-31.
11. Keshava Prasad TS, Goel R, Kandasamy K, Keerthikumar S, Kumar S, Mathivanan S, Telikicherla D, Raju R, Shafreen B, Venugopal A, Balakrishnan L, Marimuthu A, Banerjee S, Somanathan DS, Sebastian A, Rani S, Ray S, Harrys Kishore CJ, Kanth S, Ahmed M, Kashyap MK, Mohmood R, Ramachandra YL, Krishna V, Rahiman BA, Mohan S, Ranganathan P, Ramabadran S, Chaerkady R, Pandey A. Human Protein Reference Database – 2009 update. Nucleic Acids Res. 2009; 37:D767-72.
12. Ceol A, Chatr Aryamontri A, Licata L, Peluso D, Briganti L, Perfetto L, Castagnoli L, Cesareni G. MINT, the molecular interaction database: 2009 update. Nucleic Acids Res. 2010; 38:D532-9.
13. Lehne B, Schlitt T. Protein-protein interaction databases: keeping up with growing interactomes. Hum Genomics. 2009; 3:291-7.
14. Beuming T, Skrabanek L, Niv MY, Mukherjee P, Weinstein H. PDZBase: a protein-protein interaction database for PDZdomains. Bioinformatics. 2005; 21:827-8.
15. Wang Y, Liu Z, Li C, Li D, Ouyang Y, Yu J, Guo S, He F, Wang W. Drug target prediction based on the herbs components: the study on the multitargets pharmacological mechanism of qishenkeli acting on the coronary heart disease. Evid Based Complement Alternat Med. 2012; 2012:698531.
16. Wuchty S, Almaas E. Evolutionary cores of domain co-occurrence networks. BMC Evol Biol. 2005; 5:24.
17. Qi Y, Li S, Pi Z, Song F, Lin N, Liu S, Liu Z. Chemical profiling of Wu-tou decoction by UPLC-Q-TOF-MS. Talanta. 2014; 118:21-9.
18. Brenner M, Meng HC, Yarlett NC, Griffiths MM, Remmers EF, Wilder RL, Gulko PS. The non-MHC quantitative trait locus Cia10 contains a major arthritis gene and regulates disease severity, pannus formation and joint damage. Arthritis Rheum. 2005; 52:322-32.
19. Cai X, Zhou H, Wong YF, Xie Y, Liu ZQ, Jiang ZH, Bian ZX, Xu HX, Liu L. Suppression of the onset and progression of collagen-induced arthritis in rats by QFGJS, a preparation from an anti-arthritic Chinese herbal formula. J Ethnopharmacol. 2007; 110:39-48.
20. Esser RE, Hildebrand AR, Angelo RA, Watts LM, Murphey MD, Baugh LE. Measurement of radiographic changes in adjuvant-induced arthritis in rats by quantitative image analysis. Arthritis Rheum. 1995; 38:129-38.

SUPPLEMENTARY FIGURE AND TABLES

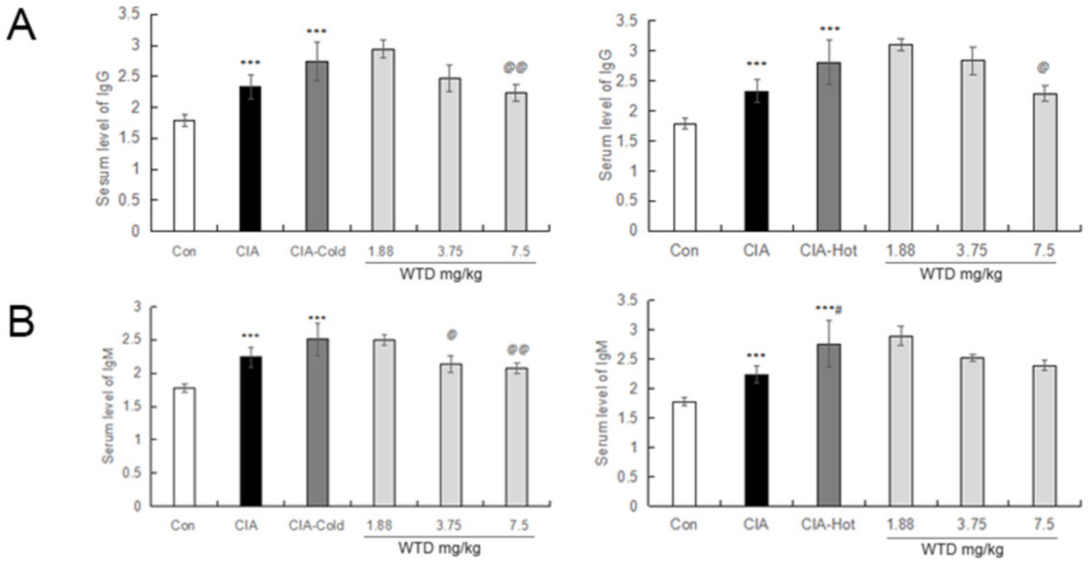

Supplementary Figure S1: Figure 6 Effects of Wu-tou decoction (WTD) on the serum levels of IgG and IgM in collagen-induced arthritis (CIA) rats. A. Serum levels of IgG in CIA-cold/hot groups were increased compared with the control group, doses of 7.5 g/(kg•day) WTD significantly lowered the serum levels of IgG in both the CIA-cold/hot model groups; **B.** Serum levels of IgM in CIA-cold/hot groups were increased compared with the control group, doses of 3.75 and 7.5 g/(kg•day) WTD distinctly decreased the serum levels of IgM in the CIA-cold groups, while this decreasing tendency did not show a statistical significance in the CIA-hot groups. Data are represented as the mean S.D (n=16). *, **, and ***, P<0.05, P<0.01, and P<0.001, comparison with the control group. #, ##, ###, P<0.05, P<0.01, and P<0.001, comparison with the CIA model group. @, @@, @@@, P<0.05, P<0.01, and P<0.001, comparison with the CIA-cold/hot model groups.

Supplementary Table S1: Putative targets of WTD predicted using the drugCIPHER-CS.

See Supplementary File 1

Supplementary Table S2: Topological features of 74 key nodes in the network based on the direct interactions between putative targets and known RA-related targets.

See Supplementary File 2

Supplementary Table S3: Composite compounds of each ingredient in WTD.

See Supplementary File 3

Supplementary Table S4: Known RA-related targets.

See Supplementary File 4

Supplementary Table S5: Detailed information on eight existing protein-protein interaction databases.

See Supplementary File 5
